# Long Term Follow-Up Safety and Effectiveness of Myopia Refractive Surgery

**DOI:** 10.3390/ijerph17238729

**Published:** 2020-11-24

**Authors:** Gracia Castro-Luna, Diana Jiménez-Rodríguez, Antonio Pérez-Rueda, Hazem Alaskar-Alani

**Affiliations:** 1 Department of Nursing, Physiotherapy and Medicine, The University of Almeria, 04120 Almeria, Spain; djr239@ual.es; 2 Department of Ophthalmology, University Hospital Torrecardenas, 04009 Almeria, Spain; a.perezrueda.oft@gmail.com; 3 Department of Ophthalmology, Hospital de Poniente, 04700 Almeria, Spain; hazemalaskar@yahoo.es

**Keywords:** myopia, laser surgery, cornea, Femto-LASIK, PRK

## Abstract

(1) Background: Refractive surgery is an increasingly popular procedure for decreasing spectacle or contact lens dependency. The two most common surgical techniques to correct myopia are photorefractive keratectomy (PRK) and femtosecond laser-assisted in situ keratomileusis (FS-LASIK). This study demonstrates the long-term effectiveness, safety, and predictability of both techniques for the refractive surgery of myopia. (2) Methods: A retrospective non-randomized study was performed. We followed 509 PRK eyes and 310 FS-LASIK surgeries for ten years. Patients were followed-up after 3 months and after 1, 2, 5, and 10 years, gathering data on their uncorrected visual acuity (UCVA) and best-corrected visual acuity (BCVA). The safety index of both procedures was defined as the quotient between the postoperative BCVA and the preoperative BCVA. We defined a procedure as safe if this quotient was equal to or greater than 1. The effectiveness index was calculated as the quotient between postoperative UCVA divided by the preoperative BCVA. (3) Results: The safety index was higher than 1 (1.09) and an effectiveness index of 0.82 after ten years of surgery in both groups was found. (4) Conclusion: These data demonstrated excellent safety and effectiveness indices for both techniques, although FS-LASIK is a technique with better safety and effectiveness indices than PRK.

## 1. Introduction

The corneal laser surgeries to eliminate myopia are currently photorefractive keratectomy (PRK) plus mitomycin C application [1,2,3,4,5,6,7,8,9,10,11] and femtosecond laser-assisted in situ keratomileusis (FS-LASIK) [12,13,14,15]. PRK is considered the first generation in the correction of refractive defects with a laser. It is performed through the use of an excimer laser, which can remove corneal tissue with microscopic precision to an exact depth with minimal alteration of the surrounding tissue. The technique consists of first removing the epithelium of the cornea, and then applying the laser that will ablate the corneal tissue necessary to correct the desired refraction defect. The procedure takes between 30 and 60 seconds. Once the surgery has been performed, a therapeutic contact lens needs to be placed for five days, which serves as an eye bandage to reduce the discomfort of the treated cornea.

In 1991, LASIK was officially introduced. It represented a new advance in refractive surgery, perfecting the previous PRK procedure. Today, it is the most widely used refractive technique. It consists of making a very thin, circular superficial cut or flap in the cornea, either by a manual cutting system with a microkeratome (blade) or using a femtosecond laser. Afterward, the corneal tissue is molded by the excimer laser, and the flap is placed in its original position. The manual system is called LASIK, and when the femtosecond laser is used, femtosecond-LASIK (FS-LASIK).

The number of FS-LASIK procedures has increased and surpasses the number of PRK procedures owing to faster visual recovery, less pain, and better ametropic range capability compared to following the former procedure [16,17,18]. PRK is still performed today, especially in corneas with superficial scarring, for epithelial dystrophies or recurrent erosions, in thin corneas, after penetrating keratoplasty, and for refractive retreatments.

Continued analysis of the safety and effectiveness of these procedures is highly relevant [18] and necessary to informed consent and evidence-based clinical practice in refractive surgery. We conducted a retrospective non-randomized analysis of the outcomes to determine the safety, predictability, and effectiveness of performing PRK or FS-LASIK after ten years.

## 2. Materials and Methods

We performed a retrospective study of 509 eyes treated by PRK and 310 treated by FS-LASIK. Data were obtained from the medical records of patients operated on from 2008 to 2019 in Invision (Almería, Spain). The ethics committee (CEI Hospital Torrecardenas) protocol number was 19/2019. The spherical equivalent refraction was between −0.5 and −23 diopters. The preoperative requirements included myopia and compound myopic astigmatism, no contact lenses for two weeks before surgery, and stable refraction for at least six months before surgery. Residual corneal bed after excimer laser ablation should be at least 300 microns without topographic signs of keratoconus. The surgery exclusion criteria included evidence of ectasia or suspected keratoconus evidenced in corneal topography, estimated postoperative corneal thickness less than 350 µm, eye disease or active systemic disease affecting corneal healing, pregnancy, and lactation. Visual acuity was measured with a standard Snellen 38 acuity chart at 6 m expressed in a decimal fraction and a logMAR(logarithm of minimum angle of resolution) scale calculated as log (MAR) = log (1/V) = −log (V). Uncorrected visual acuity (UCVA) and best corrected visual acuity (BCVA) were evaluated in all patients with an autorefractometer (ARK-700, Nidek, Japan). The following tests were performed: a biomicroscopic examination (BQ 900, Haag Strait, Swiss), an IOP(Intraocular Pressure) (noncontact tonometer, Reichert Inc., Buffalo, NY, USA), a fundus examination, a corneal topography (CM02, CSO, Oftaltech, Italy), an endothelial cell count (SP-2000, Topcon, Japan), and a pupil size test (Pupilographer, Florence, Italy). Patients did not have any ocular disease.

The ablation procedure was performed through an Esiris excimer laser (Schwind Eyetech-solution GmbH, Kleinostheim, Germany) in all the procedures in both groups. In the FS-LASIK group, the corneal flap was created with an Intralase® femtosecond laser (Intralase® femtosecond laser Abbott Laboratories Lake Bluff, Illinois, USA) before the ablation phase, taking data including depth of ablation, the thickness of the programmed flap, sphere, and cylinder programmed in the laser. Patients were followed up after 3 months and after 1, 2, 5, and 10 years, gathering data on the UCVA, BCVA, postoperative subjective refraction, topographic cylinder, and pachymetry. The safety index of both procedures is defined as the quotient of the postoperative BCVA divided by the preoperative BCVA [11,12,13]. We defined a procedure as safe if this quotient was equal or greater than 1.

The predictability was calculated as the difference between the expected spherical equivalent and the achieved spherical equivalent. The percentage of eyes with a spherical equivalent of ±1 dp (diopter) after surgery was analyzed. The efficacy index was calculated as the quotient of the postoperative UCVA divided by the preoperative BCVA [11,12,13,14]. The conditions for re-treating a patient included some of the following three parameters: a shift from an emmetropy greater than 1.00 D, a UCVA of 20/40 (<0.5 decimal fraction scale) or less and a patient’s dissatisfaction with the visual result. Undercorrection was defined as residual refraction of a spherical equivalent of −1.00 D or higher at the postoperative visit at three months. Regression was defined as a shift from an emmetropy greater than 0.5 D between follow-up visits in patients who had not undergone a retreatment.

### Data Analysis

Statistical analysis was performed using SPSS 22.0 software for Windows (SPSS Inc., Chicago, IL, USA). Values are presented as means ± standard deviation (SD); the level of significance was set at α = 0.05 (two-tailed). Normality was verified using the Kolmogorov–Smirnov test. Visual outcomes were compared using a non-parametric Wilcoxon test (two independent samples) and the Kruskal–Wallis test.

## 3. Results

Table 1 shows the demographic characteristics of both groups. No statistically significant differences were observed between the PRK group and the FS-LASIK group. The PRK patients comprised 123 men and 131 women with an average age of 31.6 ± 9.34 years. The FS-LASIK patients comprised 68 men and 87 women with an average age of 33.6 ± 10.07 years.

The mean preoperative spherical equivalent refraction was –7.18 ± 1.13 for the PRK eyes and −7.19 ± 1.06 for the FS-LASIK eyes. The preoperative BCVA mean was 0.73 ± 0.20 for PRK eyes and 0.74 ± 0.21 for FS-LASIK eyes. There were statistically significant differences in the number of retreatments (*p* < 0.05). The number of retreatments was 47 out of a total of 310 cases (15.16%) in FS-LASIK patients. The number of PRK retreatments was 125 out of a total of 509 eyes (24.55%). The reasons for retreatment were uncorrected refractive errors in all cases of FS-LASIK surgery. In the patients who underwent retreatment after PRK surgery, the reasons were a regression of refraction in 35% and uncorrected refractive errors in the rest. These regressions were probably due to the non-application of mitomycin C in all cases of PRK below −6 diopters, as reflected in the protocols of surgery at the beginning of the realization of this database. No long-term complications have been reported in PRK and FS-LASIK patients.

The spherical equivalent in the PRK group was −0.84 ± 1.01 at 3 months, −0.72 ± 0.91 at 1 year, −0.69 ± 0.90 at 2 years, −0.80 ± 1.19 at 5 years, and −1.22 ± 1.54 at 10 years after surgery. The spherical equivalent in the FS-LASIK group was −1.66 ± 1.64 at 3 months, −1.09 ± 1.22 at 1 year, −0.82 ± 1.14 at 2 years, −0.79 ± 0.83 at 5 years, and −0.92 ± 0.93 at 10 years after surgery.

### 3.1. Predictability Index

Predictability values were calculated for each follow-up period, including all eyes that were retreated, to assess the outcome of the techniques. The predictability index was ±1 dp in 80.9% of the cases at 3 months, 87.3% at 1 year, 88.2% at 2 years, 85.4% at 5 years, and 73.4% at 10 years after the PRK procedure. In FS-LASIK, the predictability index was 57.1% at 3 months, 80% at 1 year, 93% at 2 years, 75% at 5 years, and 76.2 % at 10 years after surgery (Table 2).

### 3.2. Safety Index

The safety indices at 3 months, 1 year, 2 years, 5 years, and 10 years after surgery are shown in Table 3. The preoperative BCVA was 0.73 ± 0.20 (0.1 ± 0.7 logMAR) in the PRK group and 0.74 ± 0.21 (0.1 ± 0.7 logMAR) in the FS-LASIK group. For all patients who underwent PRK surgery, the BCVA was 0.62 ± 0.18 (0.2 ± 0.7 logMAR) at 3 months, 0.70 ± 0.20 (0.1 ± 0.7 logMAR) at 1 year, 0.72 ± 0.18 (0.1± 0.7 logMAR) at 2 years, 0.77 ± 0.19 (0.1 ± 0.7 logMAR) at 5 years, and 0.79 ± 0.20 (0.1 ± 0.7 logMAR) at 10 years. For all patients undergoing FS-LASIK surgery, the BCVA was 0.74 ± 0.21 (0.1 ± 0.7 logMAR) at 3 months, 0.75 ± 0.21 (0.1 ± 0.7 logMAR) at 1 year, 0.78 ± 0.21(0.1 ± 0.7 logMAR) at 2 years, 0.80 ± 0.20 (0.1 ± 0.7 logMAR) at 5 years, and 0.82 ± 0.23 (0.1 ± 0.6 logMAR) at 10 years after surgery.

Table 4 shows the safety indices according to the surgical technique and the preoperative spherical equivalent. In all cases, the safety index is higher than 1 (meaning postoperative BCVA is almost the same as preoperative BCVA) except in PRK patients operated at 3 months and 1 year after surgery. There were significant differences between the FS-LASIK and the PRK groups. FS-LASIK was found to be the safest technique.

### 3.3. Effectiveness Index

The effectiveness index is defined as the quotient of the postoperative UCVA divided by the preoperative BCVA after surgery. UCVA was 0.58 ± 0.23 (0.2 ± 0.6 logMAR) at 3 months, 0.61 ± 0.23 (0.2 ± 0.6 logMAR) at 1 year, 0.65 ± 0.24 (0.2 ± 0.6 logMAR) at 2 years, 0.61 ± 0.27 (0.2± 0.6 logMAR) at 5 years, and 0.49 ± 0.22(0.3 ± 0.7 logMAR) at 10 years for all patients undergoing PRK surgery. UCVA was 0.63 ± 0.24 (0.2± 0.6 logMAR) at 3 months, 0.68 ± 0.25 (0.2 ± 0.6 logMAR) at 1 year, 0.67 ± 0.23 (0.2 ± 0.6 logMAR) at 2 years, 0.68 ± 0.25 (0.2 ± 0.6 logMAR) at 5 years and 0.59 ± 0.27 (0.2 ± 0.6 logMAR) at 10 years for all patients undergoing FS-LASIK surgery. The effectiveness indices were higher with a statistical difference for FS-LASIK surgery at the 3 month, 1 year, and 2 year follow-up periods. At 5 and 10 years after surgery, these differences were not maintained and the effectiveness results were equal but for a slight difference in favor of FS-LASIK surgery (Table 5).

Table 6 shows the effectiveness indices according to the surgical technique and the preoperative spherical equivalent. We found statistically significant differences between the FS-LASIK and the PRK groups. FS-LASIK was the most effective technique. However, at 5 and 10 years after surgery in the myopic range of −6 to −10 diopters and greater than −10 diopters, the efficacy rates were found to be similar for both techniques.

## 4. Discussion

Excimer laser refractive surgery has become a popular technique. New excimer lasers with aspherical ablation profiles and faster tracking mechanisms have improved the effectiveness, predictability and safety of refractive surgery. The widespread use of the femtosecond laser to create the corneal flap has been one of the most significant advances in improving the safety and reliability of the procedure compared to the mechanical microkeratome [14,15,16]. PRK is currently used in patients with suspected pathological topographies or cases of thin corneas. The use of mitomycin C on the stromal bed after ablation has allowed PRK surgeries in patients with high myopia by avoiding the appearance of haze in the postoperative period [8,9,17]. Less postoperative pain, fast visual recovery, and minimal incidence of haze has made FS-LASIK the preferred procedure in refractive surgery [18].

We performed a retrospective non-randomized study of 509 PRK eyes and 310 FS-LASIK eyes. The patients were followed for ten years. There are few publications that have gathered such a long-term follow-up of both surgical techniques. This study demonstrated good results for both long-term techniques for all preoperative spheres. The predictability of PRK surgery was higher than FS-LASIK surgery. The level of safety and effectiveness compared to FS-LASIK surgery were slighty lower. The high number of retreatments led us to consider the efficacy and safety indices divided into two groups of patients, one that underwent retreatment and one that did not [19]. The results showed a statistically significant increase in safety for patients operated with FS-LASIK compared to those operated with PRK in both the retreatment and non-retreatment groups. Comparing patient groups who underwent treatment with those who did not, found statistically significant differences in the safety indices in favor of the FS-LASIK technique except at 5 years follow-up after surgery. The effectiveness rates were higher with a statistical difference for FS-LASIK surgery at the 3 months, 1 year and 2 years follow-up periods. At 5 and 10 years after surgery, these differences were not maintained, and the efficacy results were similar but with a slight difference in favor of FS-LASIK surgery. Sajjadi et al. [20] and Al Mahmoud et al. [21] reported the same results but in shorter follow-up time after surgery. Hashemi et al. [22], after 6 months of follow-up, reported an efficacy index of 1.01 ± 0.05 for PRK and 1.01 ± 0.14 for FS-LASIK, which is higher than our results. Finally, in a recent meta-analysis of refractive surgery techniques conducted by Wen et al. [23], FS-LASIK surgery achieved the best safety and efficacy indices, although these differences were not statistically significant [24].

Currently, there is another technique called ReLEx SMILE^®^ (Carl Zeiss, 73447 Oberkochen, Germany) (small incision lenticule extraction), which began to be used in 2008 for the treatment of myopia and astigmatism. It is currently considered a minimally invasive technique. With this technique, the ophthalmologist uses only the femtosecond laser, creating a lens within the intact cornea, the thickness of which is determined by the patient’s diopters. The lenticulum is extracted through a 2 mm micro-incision, thus avoiding the circular 20 mm cut of the previous technique. The main difference of this technique with respect to its predecessors is that by not acting on the superficial layers of the cornea, it maintains the integrity of the previous structure of the cornea, avoiding possible complications associated with other techniques such as the displacement of the flap or the appearance of dry eye. This technique’s main disadvantage is that it does not allow for retreatments. If the intervention does not achieve the desired result, the patient requires PRK, LASIK, or FS-LASIK surgeries to improve the result. Despite achieving results similar to FS-LASIK [25], this technique is usually more expensive. Zhang et al. [25] concluded that SMILE and FS-LASIK are comparable in terms of both safety and efficacy.

## 5. Conclusions

All results suggest that both PRK and FS-LASIK techniques are safe and effective in the long term. However, FS-LASIK is a technique with a safety and efficacy superior to PRK. PRK surgery should be used in cases of thin corneal thickness or for topographic alterations that contraindicate the use of the FS-LASIK technique.

## Figures and Tables

**Table 1:** Preoperative characteristics of both groups.

Preoperative Characteristics	PRK	FL
Age	31.5 ± 9.6	34.2 ± 10.1
Sphere *	−7.07 ± 1.24	−7.30 ± 1.12
Cylinder *	−1.33 ± 1.07	−1.52 ± 1.11
SE	−7.78 ± 1.20	−8.08 ± 1.19
Pachymetry **	534.20 ± 38.38	539.36 ± 37.05

* diopters, SE = spherical equivalent ** microns. PRK= Photorefractive Keratectomy, FL= femtosecond laser-assisted in situ keratomileusis.

**Table 2:** Predictability index.

Techniques	3 Months *	1 Year	2 Years	5 Years *	10 Years
**Predictability** **±** **1D (%)**					
PRK	80.9%	87.3%	88.2%	85.4%	73.4%
FL	57.1%	80.0%	93.8%	75.0%	76.2%
**Predictability** **±** **2D (%)**					
PRK	93.5%	95.8%	95.2%	95.1%	89.6%
FL	85.7%	95.0%	100.0%	95.0%	85.7%

* *p* < 0.05 statistical significance.

**Table 3:** Safety index.

Techniques	3 Months	1 Year	2 Years	5 Years	10 Years
Retreatments					
**PRK**	0.88 ± 0.28	0.96 ± 0.27	1.00 ± 0.26	1.08 ± 0.28	1.11 ± 0.34
**FL**	1.04 ± 0.35	1.13 ± 0.39	1.18 ± 0.37	1.10 ± 0.42	1.21 ± 0.47
***p*-value**	<0.01 *	<0.01 *	<0.01 *	0.299	0.023
No Retreatments					
**PRK**	0.92 ± 0.29	1.04 ± 0.31	1.05 ± 0.33	1.10 ± 0.30	1.12 ± 0.34
**FL**	1.09 ± 0.32	1.15 ± 0.36	1.21 ± 0.48	1.19 ± 0.43	1.23 ± 0.49
***p*-value**	<0.01 *	<0.01 *	<0.01 *	<0.01 *	<0.01 *

* *p* < 0.05 significance.

**Table 4:** Safety index according to spherical equivalent classification.

Safety 10 y follow-up	FS-LASIK		PRK		
	Mean	SD	Mean	SD	*p*-value
Less than −10 dp	1.27	0.57	1.23	0.52	<0.05 *
−10 to −6 dp	1.19	0.34	1.12	0.33	
More than −6 dp	1.27	0.85	1.08	0.28	
**Safety 5 y follow-up**	**FS-LASIK**		**PRK**		
	Mean	SD	Mean	SD	*p*-value
Less than −10 dp	1.22	0.49	1.16	0.64	<0.05 *
−10 to −6 dp	1.14	0.31	1.08	0.29	
More than −6 dp	2.12	2.61	1.07	0.24	
**Safety 2 y follow-up**	**FS-LASIK**		**PRK**		
	Mean	SD	Mean	SD	*p*-value
Less than −10 dp	1.55	3.67	1.21	0.56	0.1
−10 to −6 dp	1.12	0.32	1.02	0.25	
More than −6 dp	1.79	3.11	1.05	0.94	
**Safety 1 y follow-up**	**FS-LASIK**		**PRK**		
	Mean	SD	Mean	SD	*p*-value
Less than −10 dp	1.19	0.4	1.16	0.53	0.06
−10 to −6 dp	1.06	0.32	0.98	0.25	
More than −6 dp	1.01	0.24	0.98	0.23	
**Safety 3 m follow-up**	**FS-LASIK**		**PRK**		
	Mean	SD	Mean	SD	*p*-value
Less than −10 dp	1.11	0.36	1	0.49	<0.01 *
−10 to −6 dp	1.04	0.28	0.87	0.24	

* *p* < 0.05 statistical significance, y = years, m = months.

**Table 5:** Effectiveness index.

Techniques	3 Months	1 Year	2 Years	5 Years	10 Years
Retreatments					
PRK	0.56 ± 0.29	0.73 ± 0.30	0.79 ± 0.32	0.87 ± 0.31	0.83 ± 0.38
FL	0.59 ± 0.35	0.86 ± 0.43	0.92 ± 0.39	0.91 ± 0.44	0.88 ± 0.49
*p* value	0.711	0.0323 *	0.0263 *	0.7854	0.5324
No Retreatments					
PRK	0.83 ± 0.29	0.92 ± 0.30	0.92 ± 0.26	0.91 ± 0.32	0.86 ± 0.44
FL	0.93 ± 0.33	0.98 ± 0.33	1.02 ± 0.51	0.94 ± 0.38	0.88 ± 0.49
*p*-value	<0.01 *	0.0281 *	0.0259 *	0.6251	0.9935

* *p* < 0.05 statistical significance.

**Table 6:** Effectiveness index according to spherical equivalent.

Effectiveness 10 y	FS-LASIK		PRK		
	Mean	SD	Mean	SD	*p*-value
Less than −10 dp	0.81	0.47	0.83	0.50	<0.05 *
−10 to −6 dp	0.97	0.42	0.82	0.39	
More than −6 dp	1.04	0.95	0.85	0.32	
**Effectiveness 5 y**	**FS-LASIK**		**PRK**		
	Mean	SD	Mean	SD	*p*-value
Less than −10 dp	0.94	0.43	0.96	0.39	0.38
−10 to −6 dp	0.92	0.38	0.91	0.32	
More than −6 dp	0.98	0.21	0.86	0.31	
**Effectiveness 2 y**	**FS-LASIK**		**PRK**		
	Mean	SD	Mean	SD	*p*-value
Less than −10 dp	1.02	0.80	0.91	0.43	<0.01 *
−10 to −6 dp	0.96	0.34	0.85	0.29	
More than −6 dp	1.28	0.31	0.86	0.26	
**Effectiveness 1 y**	**FS-LASIK**		**PRK**		
	Mean	SD	Mean	SD	*p*-value
Less than −10 dp	0.97	0.39	0.90	0.48	<0.01 *
−10 to −6 dp	0.91	0.33	0.81	0.31	
More than −6 dp	1.09	0.93	0.83	0.26	
**Effectiveness 3m**	**FS-LASIK**		**PRK**		
	Mean	SD	Mean	SD	*p*-value
Less than −10 dp	0.84	0.40	0.71	0.46	<0.05 *
−10 to −6 dp	0.82	0.32	0.69	0.30	
More than −6 dp	1.08	0.88	0.72	0.31	

* *p* < 0.05 statistical significance, y= years, m=months.
